# Is allostery a fuzzy concept?

**DOI:** 10.1002/2211-5463.13794

**Published:** 2024-05-23

**Authors:** Veronica Morea, Francesco Angelucci, Andrea Bellelli

**Affiliations:** ^1^ Institute of Molecular Biology and Pathology, CNR Rome Italy; ^2^ Department of Life, Health, and Environmental Sciences University of L'Aquila Italy; ^3^ Department of Biochemical Sciences “A. Rossi Fanelli” Sapienza University of Rome Italy

**Keywords:** allostery, arginine repressor, KNF model, MWC model, phosphoglycerate dehydrogenase, uracil phosphoribosyltransferase

## Abstract

Allostery is an important property of biological macromolecules which regulates diverse biological functions such as catalysis, signal transduction, transport, and molecular recognition. However, the concept was expressed using two different definitions by J. Monod and, over time, more have been added by different authors, making it fuzzy. Here, we reviewed the different meanings of allostery in the current literature and found that it has been used to indicate that the function of a protein is regulated by heterotropic ligands, and/or that the binding of ligands and substrates presents homotropic positive or negative cooperativity, whatever the hypothesized or demonstrated reaction mechanism might be. Thus, proteins defined to be allosteric include not only those that obey the two‐state concerted model, but also those that obey different reaction mechanisms such as ligand‐induced fit, possibly coupled to sequential structure changes, and ligand‐linked dissociation‐association. Since each reaction mechanism requires its own mathematical description and is defined by it, there are many possible ‘allosteries’. This lack of clarity is made even fuzzier by the fact that the reaction mechanism is often assigned imprecisely and/or implicitly in the absence of the necessary experimental evidence. In this review, we examine a list of proteins that have been defined to be allosteric and attempt to assign a reaction mechanism to as many as possible.

## Graphical abstract

Assigning a reaction mechanism to allosteric proteins can be difficult. It is also unclear how many reaction mechanisms qualify as allosteric and the criteria used for assigning such mechanisms. Here, we define these criteria, apply them to a list of allosteric proteins, and suggest that reaction mechanism assignment should be considered preliminary until the structural analysis is carried out.

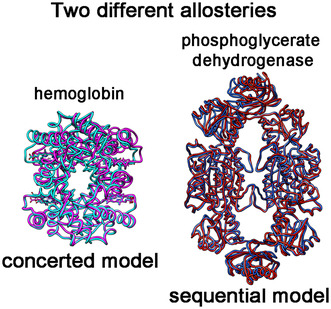

**Abbreviations**
Hb: hemoglobin
KNF: the sequential model of ligand binding of Koshland, Néméthy and Filmer (for the appropriate references, see text)
MWC: the two‐state, concerted model of ligand binding of Monod, Wyman and Changeux (for the appropriate references, see text)
PEP: phosphoenolpiruvate
PFK: phosphofructokinase
PGDH: D‐3‐phosphoglycerate dehydrogenase

## The origins of the concept of allostery

The concept and term of allostery was originally introduced by J. Monod and co‐workers in 1963 to describe the behavior of enzymes whose catalytic activity is regulated by ligands that present a chemical structure different from that of the substrate and bind to a site other than that for substrate, the interaction between the effector and the substrate being mediated by structural changes of the protein [1]. Monod and J.P. Changeux at the time were studying the catalytic properties of biosynthetic threonine deaminase, and its inhibition by isoleucine [1]. They remarked that threonine deaminase catalyzes the initial reaction of a biosynthetic pathway, and its inhibitor is the final product of the same pathway, thus realizing an elegant negative feedback regulation mechanism. This concept was born as an intuition of general applicability, but without a precise mathematical formulation; we refer to it as ‘model‐independent allostery’.

A refined structural interpretation, coupled with a rigorous mathematical formulation was proposed 2 years later by Monod, Wyman and Changeux [2], in a widely cited paper titled ‘On the nature of allosteric transitions: a plausible model’. The concerted allosteric model of Monod, Wyman and Changeux (often nicknamed MWC) postulates that allosteric proteins are symmetric oligomers that are stable in two different quaternary conformations, in free equilibrium with each other. The two conformations are envisaged as true thermodynamic states, named T (tense) and R (relaxed), which have low and high affinity for ligands, respectively. Provided that the low affinity state (T) is more populated in the absence of ligands, ligation biases the allosteric equilibrium in favor of the high affinity state (R) and causes the R state to overcome the T state. The MWC model describes positive homotropic cooperativity (i.e., cooperativity among identical ligand binding sites) and negative and/or positive heterotropic regulation (i.e., linkage between binding sites for different ligands). The ligand partition function of the MWC model of a n‐sites oligomer is as follows:

(1) $$\text{Ξ}={\left(1+\left[X\right]/K_{R}\right)}^{n}+L_{0}\;{\left(1+\left[X\right]/K_{T}\right)}^{n},$$

where *K*_R_ represents the dissociation constant of ligand X from the R state protein; *K*_T_ the dissociation constant of ligand X from the T state protein (by definition *K*_R_<<*K*_T_); and L_0_, the most characteristic parameter of the model, the equilibrium constant of the interconversion of the two states in the absence of ligand, i.e.:

(2) $$L_{0}=\left[T_{0}\right]/\left[R_{0}\right],$$

where [T_0_] and [R_0_] represent the concentrations of the protein in the unliganded T and R state, respectively, the suffix 0 indicating absence of bound ligands.

Regulatory effects occur because of changes of the allosteric constant L: the effect of ligand X is to bias L in favor of the R state:

(3) $$L_{i}=L_{0}\;{\left(K_{R}/K_{T}\right)}^{i},$$

where *i* represents the number of bound ligands and varies between 0 and *n*.

Positive homotropic cooperativity occurs when L_0_>>1 (in the absence of ligand the T state predominates), and L_*n*_<<1 (in the fully liganded protein the R state predominates). Notice that the T and R states in themselves are non‐cooperative; cooperativity occurs because of the population switch induced by ligation.

The effect of regulatory ligands different from X, and binding to other sites in the macromolecule, is to bias L_0_ in favor of the T state (for negative regulation) or in favor of the R state (for positive regulation), as follows:
(4)

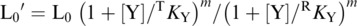

where L_0_′ is the allosteric constant in the presence of the effector Y, ^R^ *K*_Y_ and ^T^ *K*_Y_ are the dissociation equilibrium constants of Y from the R and T states respectively, and *m* is the number of binding sites for ligand Y, which may or may not equal *n*, the number of binding sites for ligand X. Eqns 3 and 4 mathematically express the concept of ‘population shift’ or ‘conformational selection’. This reaction mechanism we would like to define ‘allostery proper’, being the definitive formulation adopted by Monod and co‐workers.

The MWC model has two extremely noteworthy properties: (a) it can explain positive, but not negative, homotropic cooperativity, except under very special experimental conditions; and (b) it is concerted, i.e., it requires that all ligation intermediates, as well as the fully liganded and fully unliganded species, both for the T and R states, are symmetric, and that all subunits have the same tertiary structure and ligand affinity. Indeed, the only effect of ligands Y and X is to bias the allosteric equilibrium, in principle without changing the ligand binding properties of the T and R states. Actually, it has been experimentally observed in hemoglobin and other proteins obeying this reaction mechanism that binding of allosteric effectors often modifies *K*_T_ as well as L_0_.

The concept of allostery was thus born with an original sin: two definitions, both by Monod and co‐workers, one phenomenological, mostly focused on heterotropic regulation, as exemplified by the inhibition of Thr deaminase by Ile [1]; the other mechanistic, mostly focused on positive homotropic cooperativity [2], as exemplified by oxygen binding to hemoglobin. The second definition is more rigorous, as it includes a full mathematical description of the allosteric model, and more comprehensive, as it considers homotropic cooperativity as well as heterotropic regulation.

Since the formulation of the MWC model, several other models were proposed, usually to explain the results of specific experiments carried out on single proteins, most often hemoglobin, and, therefore, they usually lack the general applicability of the original MWC model [3, 4, 5, 6, 7, 8, 9]. These models maintain the fundamental hypothesis of two different symmetric quaternary structural arrangements/states, whose equilibrium is governed by an allosteric constant L in all states of ligation. These models belong to a single family of ‘two‐state models’, and in what follows we shall consider them as equivalent, for reasons that will become clear in the course of the analysis below.

## Other reaction mechanisms involving cooperativity and heterotropic regulation

Unfortunately, the phenomena described by model‐independent allostery may occur via different reaction mechanisms, conformation selection being only one among many. Alternative to the MWC model, and historically more ancient, is the sequential model initially formalized by Pauling [10], and refined by Koshland, Neméthy and Filmer (i.e., the KNF model) [11]. Importantly, sequential models were devised to explain homotropic cooperativity, and the effect of heterotropic regulation was conspicuously absent in the original formulations; indeed, Koshland never referred to his model as ‘allosteric’. Sequential models postulate that the structural changes responsible for the regulation of ligand affinity occur at the tertiary level because of a ligand‐induced fit mechanism, and as a result of pairwise interactions between contacting subunits of the oligomer. Therefore, sequential models differ from two‐state models in that: (a) they deny a ligand‐independent equilibrium between different quaternary structural conformations of the protein, even though they may admit a ligand‐independent conformational equilibrium between different tertiary structures of each subunit; (b) they postulate that partially liganded derivatives are asymmetric, liganded and unliganded subunits having different tertiary structures; and (c) they can explain both positive and negative homotropic cooperativity, depending on the nature of the intersubunit contacts. The ligand partition function of the KNF model varies depending on the hypothesized functional geometry of the oligomeric protein under consideration, i.e., on whether each subunit transmits the conformational change to one, two, or more other subunits. Thus, the ligand partition function of the KNF model should be derived by considering the number of subunits in the oligomer and their reciprocal arrangement. As an example, for a tetramer in which each subunit interacts with all other three subunits, the ligand partition function is as follows:

(5) $$\text{Ξ}=\begin{array}{l} 1+4\;K_{X}\;{K_{01}}^{3}\left[X\right]+6\;{K_{X}}^{2}\;{K_{01}}^{4}\;K_{11}\;{\left[X\right]}^{2}\\ +\;4\;{K_{X}}^{3}\;{K_{01}}^{3}\;{K_{11}}^{3}\;{\left[X\right]}^{3}+{K_{X}}^{4}\;{K_{11}}^{6}\;{\left[X\right]}^{4}, \end{array}$$

where *K*_X_ is the ‘intrinsic’ association constant of ligand X and *K*_01_ and *K*_11_ are called interaction parameters, and describe the modulation of the affinity constant due to the interaction between a liganded and an unliganded subunit (*K*_01_) and between two liganded subunits (*K*_11_). A *K*_00_ interaction parameter for pairs of unliganded subunits is not required because interaction parameters are conceived as relative terms and thus one of them (i.e., *K*_00_) can be assigned an unitary value. The difference between Eqns 5 and 1 is obvious and does not require specific comments. In Eqns (1), (2), (3), (4), (5), we respected the original formulation used by the respective authors; therefore, in Eqns (1), (2), (3), (4) we used dissociation constants, whereas in Eqn 5 we used association constants.

As for the MWC model, several variants of the KNF model can be envisaged. These variants are encompassed in the first definition of the model and usually imply considering different functional geometries of the macromolecule and adding further interaction parameters, to take into account heterotropic effects. The KNF model has been less widely used that the MWC model, essentially because of the difficulty to identify the functional geometry of the macromolecule, i.e., which intersubunit contacts transmit information on the ligation state and how, which makes the definition of functional geometry somewhat arbitrary. Indeed Pauling himself remarked that the oxygen binding isotherms of human hemoglobin could be fitted equally well by assuming a tetrahedral functional geometry (each subunit interacts with all the other three) or a square functional geometry (each subunit interacts with two others).

The KNF model can describe the same phenomena described by the MWC model, thus the two models, in spite of being different in the reaction mechanisms they postulate, present overlap in their applicability. As we remark below, all two‐state models and all sequential models have been tested on the oxygen binding isotherms of hemoglobin and on other proteins, and have usually been proved to be capable of describing cooperative ligand binding isotherms with great accuracy. This demonstrates that the ability of fitting ligand binding data is not proof of validity of a model and that more refined experiments are necessary to assign a reaction mechanism, and thus a model, to an allosteric protein.

Ligand‐linked association‐dissociation is another mechanism able to explain homotropic cooperativity in ligand binding and heterotropic regulation, for reasons analogous to those considered for the MWC model. Indeed, in this case, the protein has two states, represented by different degrees of oligomerization and, if the different oligomers have different ligand affinity, positive homotropic cooperativity will ensue [12]. Moreover, heterotropic ligands may affect the association‐dissociation constant. The difference between ligand‐linked association‐dissociation and the MWC model is that the association constant of the monomers in the former model, which plays the same role as the allosteric constant L_0_ in the latter, is not dimensionless; however, ligand‐linked association‐dissociation is a kind of population selection.

Strictly speaking, intermediate reaction mechanisms and models do not exist. Either a protein obeys a sequential mechanism or it does not; either it presents an allosteric equilibrium between two states or it does not; etc. These statements do not refer to the actual value of the parameters involved: e.g. in a protein that obeys a two‐state reaction mechanism the value of L_0_ may be very large, implying minimal population of the R_0_ state, but this does not make it to approximate a sequential reaction mechanism, since if it presents homotropic or heterotropic effects at some degree of ligation the allosteric constant, be it L_0_' or L_*i*_, must approximate unity to allow the quaternary structure switch.

Mixed reaction mechanisms are possible if the same protein presents two or even all three mechanisms together, i.e., not an intermediate mechanism but the sum of two or three. Wyman called this effect ‘nesting’ to describe a cooperative substructure embedded into a larger more cooperative superstructure. Nesting has been invoked for some O_2_ carriers [5, 6], in which the T state, besides being in equilibrium with the R state, might present sequential cooperativity. Another possibility is the association of the allosteric T–R quaternary structure equilibrium with ligand‐linked dissociation, which has been observed, for example, in hemoglobin.

The problem that we face in scientific literature is that many proteins that are classified as ‘allosteric’, because they present homotropic cooperativity and/or heterotropic regulation, obey reaction mechanisms different from the two‐state model (see Table 1 below). In other words, there is not one single mathematical definition corresponding to allostery, but many. However, since this situation is rarely acknowledged, allostery has become an imprecisely defined concept. Once this condition is acknowledged, it follows that it would be advisable to define which type of allostery each protein possesses.

**Table 1:** List of the allosteric proteins analyzed by Daily and Gray [14], with some additions (*), ordered according to the (presumed) thermodynamic mechanism. List of ‘allosteric’ proteins, integrated with our analysis of the reaction mechanism. Since neither the MWC nor the KNF models consider monomeric proteins, these are not assigned a specific reaction mechanism and are satisfactorily described by Wyman's linked functions. In some cases, the reaction mechanism is presumptive, because the available information is insufficient for a certain attribution. The proteins we added to the list by Daily and Gray [14] are marked with an asterisk, and were considered allosteric by the authors who studied them, but not necessarily because of heterotropic regulation; e.g. Furukawa *et al*. [29] consider D‐lactate dehydrogenases allosteric because they present homotropic cooperativity, although via a sequential reaction mechanism. The references quoted in the table usually report and describe the structure indicated by the pertinent PDB code(s); in some cases, further references were added that describe the functional properties of the protein. The criteria for the assignment of the reaction mechanism are described in the text. The parameter R1 cannot be determined in the cases of monomeric proteins, or ligand‐linked association‐dissociation. ‘Undefined’ indicates that the available information does not allow the identification of the reaction mechanism; ‘Not applicable’ indicates that the monomeric state of the protein is not compatible with the criteria defined in Section ‘Identification of the evidence required for mechanism assignment’. Covalent modification may cause structural and functional changes, but is not compatible with either the MWC or KNF reaction mechanism, which presume rapid and reversible chemical equilibria; however, each state a covalently modifiable protein (e.g. because of phosphorylation) may obey a KNF or MWC model, as it occurs, for example, in the case of glycogen phosphorylase.

Protein and PDB codes; references in square brackets	Thermodynamic mechanism	Notes
Hemoglobin* [13, 15] 2DN3; 2DN2	Concerted, MWC‐like, and ligand‐linked oligomerization	Heterotetramer; see Section ‘Hemoglobin’. R1 = 0.28
Asp‐transcarbamylase [13] 6AT1; 8ATC	Concerted, MWC‐like	12‐mer; demonstrated ligand‐independent T–R structure change. R1 = 0.28
Phosphofructokinase [13] 6PFK; 4PFK	Concerted, MWC‐like	Homotetramer; ligand‐independent T–R structure change is induced by effectors. R1 = 0.60
Glycogen phosphorylase [13] 1GPB; 7GPB	Probably concerted, MWC‐like; admits covalent modification (phosphorylation)	Homodimers reversibly associating to homotetramers. R1 = 0.51
Glucosamine‐6‐P deaminase [16, 17] 1CD5; 1HOT	Probably concerted, MWC‐like	Symmetric homo‐6‐mer; R1 = 0.25
Chorismate mutase [18] 2CSM; 1CSM	Possibly concerted, MWC‐like	Homodimer, activated by Trp and inhibited by Tyr. R1 = 0.68
Anthranilate synthase [19] 1I7S; 1I7Q	Possibly concerted	Biosynthesis of Trp; inhibited by Trp. A_2_B_2_ heterotetramer. R1 = 0.49
Human mitochondrial malic enzyme [20, 21, 22] 1QR6; 1PJ2	Possibly concerted	Symmetric homotetramer; oxidative decarboxylation of malate to pyruvate; positive cooperativity for malate; activation by fumarate. Stoichiometric ratio fumarate:malate = 1 : 1. R1 = 0.58
*L. casei* L‐lactate dehydrogenase [23] 2ZQY; 2ZQZ	Concerted	Cooperative homotetramer, inhibited by fructose 1,6 bisphosphate. allosteric structure change demonstrated by its pH dependence. R1 = 0.26
Transcriptional activator DctD [24] 1L5Z; 1L5Y	Probably concerted	Symmetric homodimer. R1 = 0.27
3‐phosphoglycerate dehydrogenase [25, 26] 1PSD; 1YBA	Sequential, atypical because of significant quaternary structure change	Homotetramer; Ser biosynthesis pathway; negative homotropic cooperativity for inhibitor L‐Ser. R1 = 0.56
Arg repressor (ArgR) [27, 28] 1XXC; 1XXA	Sequential	Homo 6‐mer; negative homotropic cooperativity for Arg. R1 = 0.81
*Pseudomonas aeruginosa* D‐lactate dehydrogenase* [13, 29] 6ABJ; 5Z20	Sequential	Homotetramer; negative homotropic cooperativity. R1 = 0.73
Uracil‐phosphoribosyl transferase from *S. solfataricus* [30, 31] 1XTU; 1XTT; 1XTV	Sequential	Homotetramer. Inhibited by CTP; negative homotropic cooperativity for UMP. Significant intramolecular asymmetry for 1XTV. R1 ~ 0.6
3‐deoxy‐D‐arabino‐ heptulosonate‐7‐phosphate (DAHP) synthase [32, 33, 34] 1KFL; 1GG1	Possibly sequential.	Biosynthesis of aromatic aa. Symmetric (D2) homotetramer. non‐cooperative; non‐competitively Inhibited by Phe. R1 = 0.45
*E. coli* purine nucleotide phosphorylase (PNP)* [13] and references therein; 1ECP; 4TTA	Sequential	Homo 6‐mer, dimer of trimers; negative homotropic cooperativity; strong intersubunit asymmetry. R1 = 1.05
*E. coli* Met repressor [35] 1CMB; 1CMA	Possibly sequential	Homodimer; ligand‐ induced asymmetry
dTMP synthase* [36] 1CI7	Sequential	Homodimer; negative homotropic cooperativity; no known effectors
Purine repressor PurR [37, 38] 1DBQ; 1WET	Undefined	Homodimer; symmetric when liganded to guanine and DNA; minor asymmetry when ligand‐free. R1 = 0.87
Tetracycline resistance repressor (TetR) [39] 2TRT; 2XB5; 1QPI	Undefined	Symmetric homodimer. Binds either to tetracycline or to DNA; Tc binding site at the monomer‐monomer interface. R1 = 1
Fructose bisphosphatase (FBPase‐1) [40] 1EYJ; 1EYI	Undefined; insufficient information	Homotetramer. Significant subunit asymmetry. R1 > 1
ADP‐ribosylation factor 1 (arf1) [41] 1HUR	Undefined; insufficient information	Symmetric homodimer
GTP cyclohydrolase I [42] 1WPL; 1IS7	Undefined, complex: inhibited by the biopterin‐ binding regulatory protein GFRP. Multiple aggregation states	Biosynthesis of biopterin. GTP cyclohydrolase I is a 10‐membered ring; the regulatory protein GFRP is a 5‐membered ring; the complex of the two involves one cyclohydrolase and two GFRP forming a hetero 20‐mer
Heat labile enterotoxin [43, 44] 1LTT; 1LTR	Undefined	Binds lactose; hetero 7‐mer composed by a 5‐membered ring of identical subunits plus two other subunits
ATP sulfurylase [45] 1M8P; 1I2D	Undefined	Symmetric homo 6‐mer, inhibited by 3′‐phosphoadenosine‐5′‐phosphosulfate; R1 = 0.88
*E. coli* arabinose binding protein (AraC) [46, 47] 1XJA; 2ARA; 2ARC	Ligand‐linked association‐dissociation; forms two different homodimers, depending on the presence of arabinose	Possibly higher assembly states in the absence of arabinose. Open structure in spite of isologous interfaces
G‐protein rab11 [48] 1OIV; 1OIW	Ligand‐linked oligomerization?	Quasi‐symmetric homodimer in the inactive GDP‐bound state; monomer when the GTP‐bound
Kinase domain of insulin‐like GF receptor IGRK [49, 50] 1P4O; 1K3A	Covalent modification (phosphorylation); ligand‐linked oligomerization?	Monomer when unliganded; heterodimer when liganded?
*E. coli* repressor of the biotin operon (BirA; BioR) [51, 52] 1BIA; 1HXD	Ligand‐linked oligomerization	Monomer when free, dimer when biotin‐ and DNA‐bound
Tyr kinase domain of human insulin receptor (IRK) [53] 1IRK; 1IR3	Ligand‐linked oligomerization; phosphorylation	
ATP‐phosphoribosyl transferase [54] 1NH8; 1NH7	Ligand‐linked association‐dissociation; possibly concerted	His biosynthesis; inhibited by His. Dimer when His‐free, hexamer when His‐bound
Lac repressor (LacR) [55, 56] 1TLF; 1EFA	Ligand‐linked oligomerization	Homotetramer when bound to isopropyl‐beta‐D‐thiogalactoside; homodimer when bound to DNA
Nitrogen fixation enzyme fixJ [57, 58] 1DBW; 1D5W	Ligand‐linked oligomerization; covalent modification (phosphorylation)	Monomer/dimer equilibrium
E. coli H_2_O_2_ sensor (oxyR) [59] 1I69; 1I6A	Covalent modification; redox‐dependent oligomerization (intrasubunit disulfide bridge formation)	Homodimer in the reduced state, monomer in the oxidized state
E. coli chemotaxis protein CheY [60] 3CHY; 1FQW	Covalent modification (phosphorylation)	Monomer
Protein kinase B (PKB) [61] 1GZK; 1O6K	Covalent modification (phosphorylation)	Monomer; suggested conformational equilibrium
Anti sigma factor antagonist SpoIIAA [62] 1H4Y; 1H4X	Covalent modification (phosphorylation)	Monomer
Caspase [63] 1SHJ; 1F1J	Covalent modification (disulfide formation)	Homodimer
MAP kinase ERK2 [64, 65] 5UMO (previously 1ERK); 2ERK	Covalent modification (phosphorylation)	Monomer
GTP binding protein Ran [66] 1IBR	Not applicable	Monomer
Protooncogene RAS [67] 4Q21; 6Q21	Not applicable	Monomer; activated by GTP; inhibited by GDP
GTPase cdc42 [68] 1AN0; 1NF3	Not applicable	Monomer
rac1 [69] 1HH4; 1MH1	Not applicable	Monomer
Protein Tyr phosphatase 1 B (PTP1B) [70, 71] 1T48; 1PTY	Not applicable	Monomer
GTP binding protein sec4 (Rab family) [72] 1G16; 1G17	Not applicable; possible Ligand‐linked oligomerization	Monomer
GTPase rheb [73] 1XTQ; 1XTS	Not applicable	Monomer
Elongation factor EfTu [74, 75] 1TUI; 1EFT	Not applicable	Monomer; GTP‐dependent binding to aminoacyl‐tRNA
GTPase ypt7p [76] 1KY3; 1KY2	Not applicable	Monomer
G‐protein rap2a [77] 1KAO; 2RAP	Not applicable	Monomer
GTP binding protein YsxC [78] 1SVI; 1SVW	Not applicable	Monomer
GTP binding protein arf6 [79] 1E0S; 2J5X (formerly 1HFV)	Not applicable	Monomer
Transducin alpha subunit [80, 81] 1TAG; 1TND	Not applicable	Alpha subunit of the heterotrimeric GTP binding protein transducin
rab7 [82] 1VG1; 1VG8	Not applicable	Monomer; binding of GTP/GDP regulates affinity for REP1 protein
GTPase rhoA [83, 84] 1FTN; 1A2B	Not applicable	Monomer; binds GTP/GDP
GTP hydrolase Gi [85, 86] 1GDD; 1GIA	Not applicable	Alpha subunit of heterotrimeric GTP binding protein

## Assignment of the reaction mechanism to a list of allosteric proteins

Since allostery is an appealing concept, it has been invoked for a vast number of proteins, and it is commonly used to refer to homotropic cooperativity, heterotropic regulation, or both. Moreover, as discussed above, this concept has been associated in the literature with several reaction mechanisms, in addition to the MWC model. The problem we face is thus to classify the reaction mechanisms capable of producing homotropic and heterotropic regulation and to define the criteria for assigning the appropriate mechanism.

### Identification of the evidence required for mechanism assignment

Identification of the reaction mechanism underlying allostery cannot be based on ligand binding experiments and crystallographic structures of the fully unliganded and fully liganded states alone, but requires subtle and refined experiments designed to test the following specific features: (a) whether the structure of the ligation intermediates is symmetric or asymmetric, and equal to either the fully liganded or the fully unliganded derivative; (b) whether there is evidence for ligand‐independent quaternary structure change; (c) whether the structures of the ‘unfavorable’ states hypothesized by the MWC model (i.e., liganded T and unliganded R) can be determined, together with those of the more favorable states (i.e., unliganded T and liganded R); (d) whether cooperativity and regulation depend on protein concentration (which suggests ligand‐linked association‐dissociation); and (e) whether the protein presents negative homotropic cooperativity, which is incompatible with population selection, and is thus proof of a sequential model (some very uncommon exceptions may occur). Thus, the reaction mechanism of a ligand binding protein can be identified as MWC‐like if it obeys any of the following criteria: symmetry of ligation intermediates; evidence of the ligand‐independent (quaternary) structure change; evidence for the existence of the unfavorable states. A ligand binding protein can be assumed to obey a KNF reaction scheme if it presents significant intersubunit asymmetry, especially in the incompletely saturated ligation intermediates or negative homotropic cooperativity. Finally, a protein can be assigned a ligand‐linked association‐dissociation mechanism if it presents evidence of concentration‐dependent changes in ligand affinity. The information required for assignment of the reaction mechanism may not be always available, in which case the assignment should be considered presumptive or provisional.

In addition to the experimental criteria listed above, we also examined a structural parameter recently identified by ourselves based on the analysis of the 3D structures of the liganded and unliganded state of some allosteric proteins whose reaction mechanism could be assigned with certainty [13]. Following this analysis, we found that, as expected, the MWC‐like proteins present small ligand‐dependent tertiary structure changes coupled to large quaternary structure changes, whereas the opposite occurs for KNF‐like proteins [13]. We measured the RMSD values between the α‐carbons of liganded and unliganded structures and called R1 the ratio between the RMSD values measured for the isolated subunits and the RMSD values measured for the whole oligomers. We found that R1 values are < 0.3 in typical two‐state, MWC‐like proteins and > 0.7 in typical sequential, KNF‐like proteins (see Table 1). In the case of strong but ordered intersubunit asymmetry, R1 may exceed unity. Intermediate values are observed when the protein obeys mixed reaction mechanisms (see above) or when the protein follows a sequential, KNF‐like model and the ligand‐induced structural changes are very small at both the tertiary and quaternary level. The R1 parameter is not a clear‐cut proof of the reaction mechanism, but it may be taken as an indication when more refined information is lacking. Unfortunately, the parameter R1 cannot be determined for monomeric proteins, or for proteins that undergo ligand‐linked association‐dissociation equilibria, and it is not meaningful in the case of proteins that undergo covalent modification.

### Analysis of a list of ‘allosteric’ proteins

Table 1 lists the proteins considered allosteric by Daily and Gray [14], with the addition of some others (marked by an asterisk), considered allosteric by the authors of the papers cited in the Table. It is crucial for the scope of this review that we use a list of allosteric proteins compiled by other authors, otherwise our analysis would be biased by our views on allostery. However, we felt free to implement the original list by Daily and Gray, chosen because it is extensive, with other allosteric proteins, so defined by the authors of the pertinent papers, because Daily and Gray apparently considered allostery a synonymous of ‘presenting a heterotropically regulated structure change’, thus excluding pure homotropic cooperativity. While Daily and Gray did not try to analyze reaction mechanisms, in this work we carried out an extensive literature search for each of the proteins in the list to assign to each of them the presumed or demonstrated reaction mechanism. In some cases, we confirmed what was reported in the literature, but in others we added a missing assignment or corrected the original one. The criteria to assign the reaction mechanism are summarized above and more extensively explained in Table 1 of ref. [13].

The Table is by no means exhaustive, but is, in our opinion, representative of what researchers may define an ‘allosteric protein’, and this is what matters for our analysis. Entries are ordered according to the reaction mechanism, with the caution that any single protein may present more than one reaction mechanism: e.g., ligand‐linked oligomerization may coexist with concerted cooperativity, or with covalent modification. Clearly, allosteric proteins share neither the reaction mechanism nor the aggregation state, and the only property the entries of Table 1 have in common is that they bind ligands and present homotropic and/or heterotropic regulation of ligand affinity (i.e. all of them present model‐independent allostery); moreover they present ligand‐dependent structure changes, but we suspect that no protein can bind a ligand without experiencing some structure change.

Inspection of Table 1 shows that, for each of the well‐established reaction mechanisms between proteins and ligands described in the previous paragraph, pertinent examples can be quoted; moreover, some unexpected additions are present in the Table, namely covalently regulated proteins and monomeric proteins. In the case of many entries of the Table the reaction mechanism was correctly assigned, in some cases no specific reaction mechanism was identified, or a reaction mechanism had been assigned in the absence of sufficient evidence, or was wrongly assigned. This is often the case for the two‐state MWC model that, due to its popularity, has been associated with a large number of allosteric proteins, even when insufficient information was available. As an example, whenever the fully liganded and fully unliganded structures of a ligand binding protein have been determined, the former has been assumed to pertain to the R state, and the latter to the T state. This language is misleading, as it conveys the idea that in the protein a ligand‐independent equilibrium exists between the two quaternary structures, when in fact none has been demonstrated to occur (or not to occur).

Table 1 lists 55 proteins, tentatively grouped according to their reaction mechanism as follows (to some proteins more than a single mechanism is assigned). Ten entries can be assigned to the class that obeys a concerted reaction mechanism based on population selection, hence the MWC model or any of its more recent variants. Eight entries obey a ligand‐linked oligomerization mechanism; this class is most probably underrepresented, but there is some overlap with the class of proteins that require covalent modification. As already stated, ligand‐linked oligomerization is a kind of population selection.

Eight entries of Table 1 could be more or less confidently assigned to the sequential reaction mechanism proposed by Koshland *et al*. [11]. Two of these, D‐lactate dehydrogenase and dTMP synthase, were not present in the original list by Daily and Gray, probably because they have no known heterotropic effectors; we added them because the authors of the relevant papers, explicitly call them allosteric as they present homotropic regulation of substrate affinity; thus they serve to further illustrate how subjective the definition of allostery has become.

Seven entries of Table 1 are labeled as ‘undefined’. These proteins obey either a MWC‐like or a KNF‐like reaction mechanism, but the available information is insufficient to assign the mechanism more precisely, or they present features that may be compatible with both mechanisms (e.g., a small quaternary structure change, evident as a value of R1 close to unity, coupled to minimal asymmetry in both the liganded and unliganded states).

Six entries of Table 1 refer to proteins in which a structure change is caused by covalent binding of the heterotropic effector (e.g., phosphorylation). Covalent modification, unless rapid and reversible, does not fit with either the MWC or KNF reaction mechanism, which postulate rapidly reversible equilibria, thus these proteins do not conform to Eqns (1), (2), (3), (4), (5). However, the two states of the native and covalently modified protein should be considered as two different entities, and the possibility arises that either of them obeys a MWC or KNF mechanism or presents ligand‐linked dissociation‐association. An example is glycogen phosphorylase, whose phosphorylated state seems to obey a MWC‐like reaction mechanism.

Finally, 16 entries of Table 1 refer to proteins that are monomers in solution or have been studied as monomeric components of assemblies made of different subunits. These obviously lack homotropic interactions and are allosteric only in the sense that they bind (at least) two ligands to different sites, and the affinity of either influences that of the other(s); thus, their functional behavior can be described using Wyman's linkage functions [87, 88, 89]. Monomeric proteins evade our criteria for assigning a reaction mechanism and are discussed under a separate heading (see below).

Some proteins fall in two categories (e.g., monomers and covalent modification). We may add that in some cases of covalent modification, or other types of structure changes, the protein may undergo a slow rearrangement that may mimic equilibrium cooperativity. These cases may be difficult to detect in the absence of specific kinetic information and may contaminate the mechanism assigned to some of the proteins in Table 1.

Some reaction mechanisms are probably underrepresented in Table 1. In particular, certain positive criteria for the sequential reaction mechanism are very restrictive (e.g. negative cooperativity essentially proves the mechanism, but positive cooperativity does not disprove it); thus it is likely that several ‘undefined’ entries might in fact be cases of KNF‐like reaction mechanism. However, for the purposes of the present work, an unequal representation of the possible reaction mechanisms that may be grouped under the umbrella of allostery does not invalidate the finding that allostery is a fuzzy concept, in need of some revision, or at the very least of the recognition that it groups different entities.

### Stoichiometry of ligands and effectors

The mechanism of action of heterotropic effectors in proteins that present population selection is to bias the allosteric (or oligomerization) constant. Since these proteins are oligomers whose function is regulated at the level of the quaternary structure, the binding site of the heterotropic effector is not related to the number of protomers: e.g. the O_2_ affinity of hemoglobin is regulated by CO_2_, whose stoichiometric ratio to oxygen is 1 : 1 (one site per protomer), by bezafibrate, with a ratio to oxygen of 1 : 2 (two sites per tetramer), and by glycerate bisphosphate with a ratio to oxygen of 1 : 4 (one site per tetramer). By contrast, in the proteins that obey the sequential KNF reaction scheme heterotropic regulation usually occurs at the tertiary structure level, and in all cases considered in Table 1 they possess one binding site *per* subunit.

An interesting observation is that in some proteins that obey a KNF reaction mechanism heterotropic effectors present two different stoichiometric ratios between subunits and effectors; this is observed, for example in PGDH and in the Arg repressor [13]. In these cases, the true stoichiometry of the effector is 1 per subunit, as expected for KNF proteins, but the functional regulation is fully achieved at lower stoichiometric ratios (0.5 per subunit or 2 per tetramer in the case of PGDH). It is also common that in these cases the binding of the effector presents negative cooperativity (see below). In these cases, the stoichiometry one calculates from enzyme activity differs from the one observed by directly measuring effector binding or by determining the number of bounds effectors by X‐ray crystallography.

## Some examples

The principal aim of the present review is the assignment of reaction mechanisms to the allosteric proteins listed in Table 1. Since this process is quite intricate and may not be obvious to all readers, we provide here some examples of MWC‐like and a KNF‐like cases, to practically illustrate how the criteria defined under Section ‘Identification of the evidence required for mechanism assignment’ are used. We do not provide examples of the assignment of the ligand‐linked association‐dissociation mechanism, because it is straightforward and not open to doubt.

### Hemoglobin

**Fig. 1:**
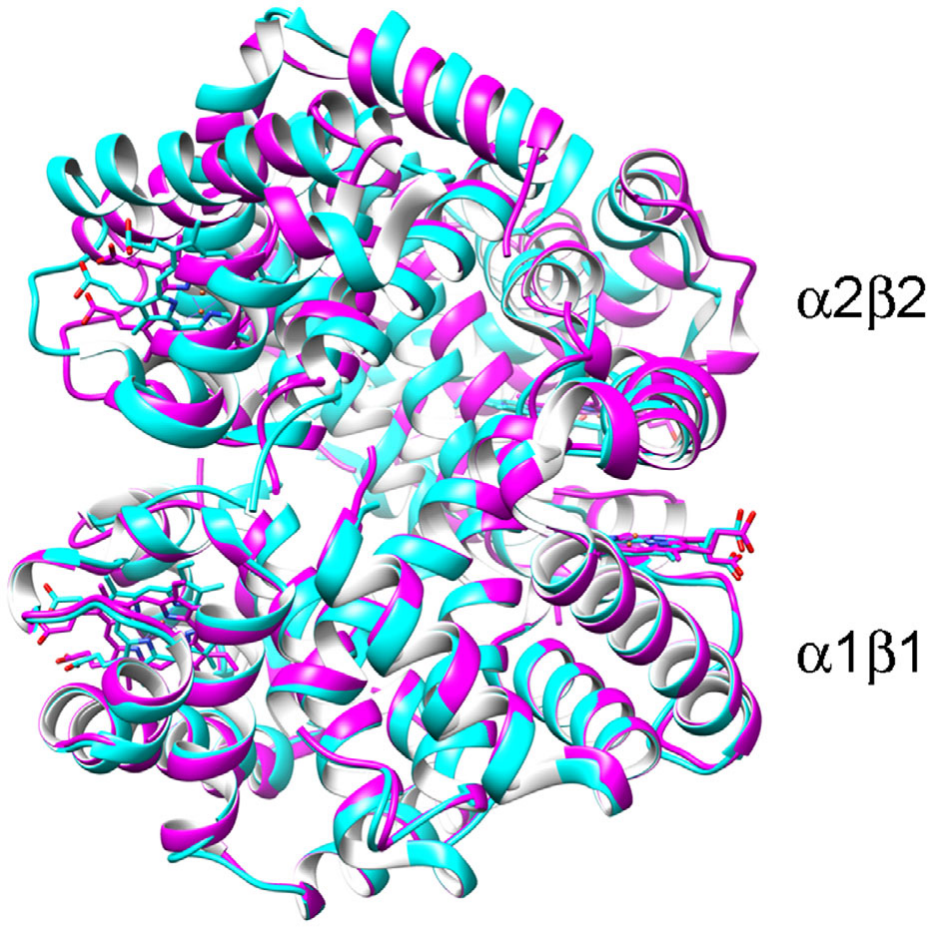
Allosteric quaternary structure transition in human hemoglobin. The quaternary structure change of human hemoglobin is described as a rotation and translation of one α1β1‐type heterodimer with respect to the other. The α1β1 heterodimers form T state (purple) and R state HbA (light blue) are superimposed, to demonstrate their different spatial relationship with the α2β2 heterodimers form T state and R state HbA. The tertiary structure changes are comparatively small, as one may notice in the superposition of the α1β1 heterodimers.

Hemoglobin is the prototype of a protein that obeys a two‐state MWC‐like reaction mechanism (see Fig. 1). The rich spectroscopic properties of the heme, the ease of preparation and crystallization, and the availability of different derivatives have allowed researchers to conduct experiments that are impossible on other proteins. Since we reviewed the allosteric properties of hemoglobin elsewhere [15], we shall not go in detail in this work, but shall list only the key pertinent features that allow the unequivocal assignment of Hb to the concerted allosteric mechanism. We also remark that several variants of the MWC model were devised to obtain a more precise description of refined experiments conducted on this protein; but in this work, we are not concerned with subtleties that would require identification criteria that cannot be met for any other protein among those listed in Table 1.

The oxygen binding isotherms of hemoglobin were very satisfactorily described using all sequential models [10, 11], and all concerted models [2, 3, 4, 5, 6, 7, 8, 9], thus demonstrating that ability to describe the ligand binding curve is not proof of the applicability of the model. The assignment of the reaction mechanism of Hb is based on the following experimental evidence:

1. The less populated states of Hb could be crystallized and solved, together with the most populated ones: i.e. we know the structures of ^T^Hb, ^T^Hb(O_2_)_4_, and ^R^Hb(O_2_)_4_ [15] and references therein. All structures are symmetric or almost so. The structure of ^R^Hb is known via its mimics, e.g., ^R^Hb reacted with BME. Thus, the ligand biases the allosteric equilibrium constant but does not determine the quaternary structure of the protein, coherently with the MWC model and its variants.
2. The allosteric structure change could be detected in real time, via the associated spectroscopic signals, in the following experiments: the modulated photoexcitation of Hb(CO)_4_‐Hb(CO)_3_; the titration of the Root effect HbIV(CO)_4_ from trout with pH; the rearrangement of Hb after photolysis [90]. Thus, not only the ligand‐independent quaternary structure equilibrium has been demonstrated, but the allosteric constants have been directly measured, at least for some derivatives.
3. The structure of doubly liganded intermediates could be solved thanks to the replacement of two iron atoms in the tetramer with Mn or Zn which form stable five‐coordinate complexes mimicking the unliganded Fe. The other two Fe atoms were CO‐liganded [15] and references therein. Doubly liganded intermediates crystallize as symmetric T state tetramers; no intermediate states were found. Actually, intermediate quaternary structures could only be observed by restricting the motion of the subunits by cross‐linking.

Thus, in the case of Hb all three criteria of MWC‐like reaction mechanism are fulfilled, and there can be no reasonable doubt on the assignment.

### Phosphofructokinase

Phosphofructokinase presents several features compatible with the concerted, MWC‐like reaction mechanism [91]: (a) it presents positive homotropic cooperativity for fructose 6‐phosphate (F6P), and heterotropic inhibition by phosphoenolpyruvate (PEP); (b) it is a homotetramer presenting isologous interfaces and may be described as a dimer of dimers; (c) the homotetramer is symmetric both in the F6P‐liganded state and the PEP‐inhibited state, and the two quaternary structures differ because of a rotation of one dimer with respect to the other, much like hemoglobin; and (d) the ligand‐induced quaternary structure change is much larger than the tertiary structure change of each subunit. Unfortunately, direct evidence of the allosteric transition at constant degree of ligation with F6P is not available; but the enzyme from *Escherichia coli* in the absence of F6P and PEP crystallizes in the putative R state, a quite surprising finding, and crystallization in the T state is only obtained in the presence of PEP [91]. One might explain this finding as evidence of an allosteric equilibrium between two states, biased by the inhibitor. Thus, it seems very plausible that PFK qualifies for a concerted, MWC‐like reaction mechanism.

### Phosphoglycerate dehydrogenase from *E. coli*

**Fig. 2:**
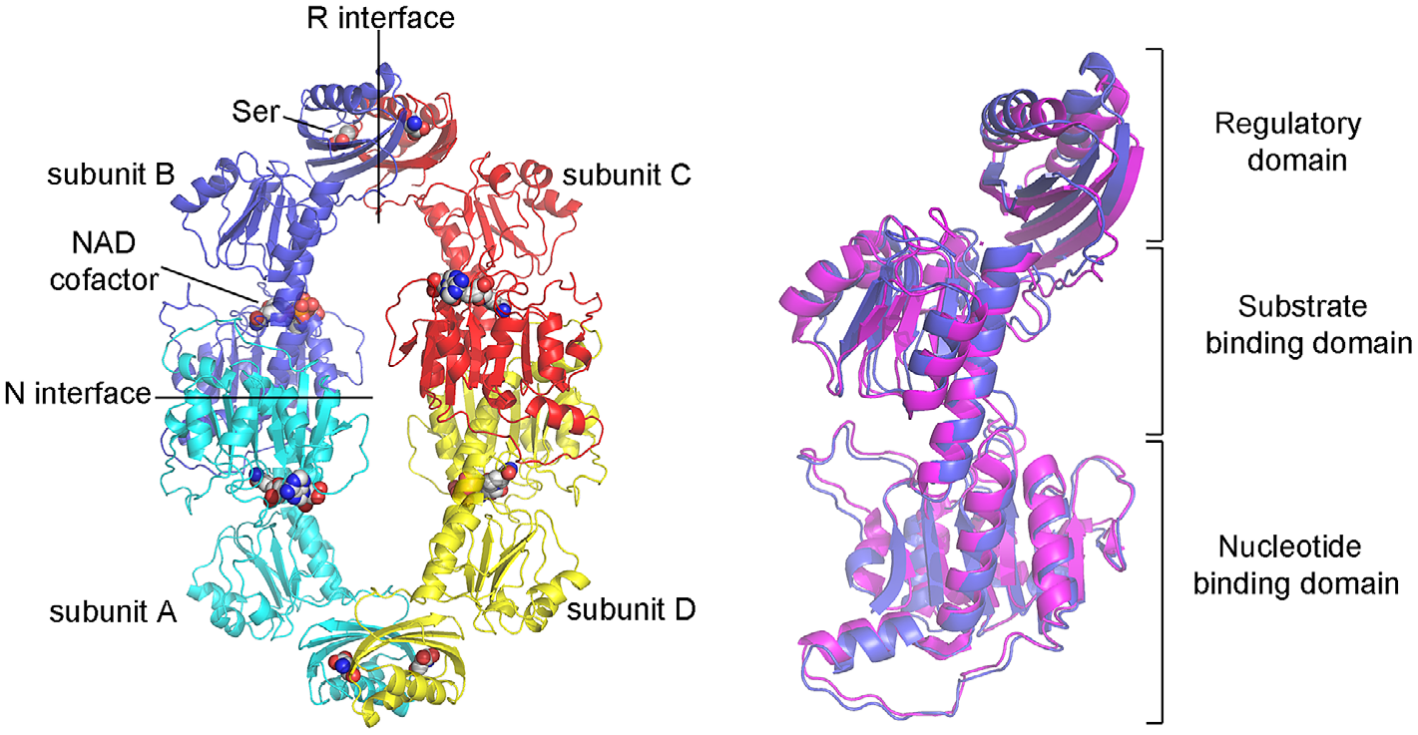
Structure of *E. coli* PGDH. Left panel: tetrameric structure of PGDH; the subunits are shown in different colors; the interfaces between regulatory domains (R interfaces) are at the top and bottom of the structure, those between NADH binding domains on the left and right corners of the rhomboidal structure (N interfaces). The substrate (NAD^+^) and inhibitor (Ser) are shown as spheres. Right panel: superposition of subunits B from Ser‐free and Ser‐bound PGDH, showing the tertiary structural changes and reorientation of the regulatory domain with respect to the nucleotide binding domain.

D‐3‐phosphoglycerate dehydrogenase (PGDH) is the enzyme that catalyzes the first step of L‐Ser biosynthesis pathway. The *E. coli* enzyme is a homotetramer that is inhibited by L‐Ser, whose binding stoichiometry is one molecule per subunit (see Fig. 2).

PGDH is assumed by some authors to obey the two‐state MWC model [25], but in our opinion this assignment should be revised, because the enzyme presents negative homotropic cooperativity with respect to both the phosphoglycerate substrate [92], and the inhibitor L‐Ser [93], a feature that cannot be accounted for by the MWC model or its variants. The structural parameters of *E. coli* PGDHs are quite compatible with the hypothesis of KNF‐like reaction mechanism: the enzyme is a homotetramer; the quaternary structure obtained in the presence of the inhibitor L‐Ser is symmetric, whereas that obtained in the presence of the substrates NAD^+^ and alpha‐ketoglutarate presents significant asymmetry. Thus, we feel confident in assigning PGDH to the KNF‐like group.

### D‐Lactate dehydrogenase

Bacterial lactate dehydrogenases can be distinguished according to their stereospecificity into D‐ and L‐LDHs. D‐ and L‐LDHs are evolutionarily unrelated and present wide divergence even within their respective families. In Table 1 there are separate entries for D‐ and L‐LDHs and it is noteworthy that, though both present homotropic cooperativity, D‐LDHs obey a KNF‐like reaction mechanism and some of them present negative homotropic cooperativity for their substrate, whereas most L‐LDHs are supposed to obey a MWC‐like mechanism and present positive homotropic cooperativity and allosteric regulation by fructose 1,6 bisphosphate. Furukawa *et al*. characterized the functional and structural properties of three D‐LDHs from Gram negative bacteria and correctly assigned their reaction mechanism as sequential, KNF‐like, because of their negative homotropic cooperativity [29]. However, these authors claimed both families of LDHs to be allosteric, because of their homotropic cooperativity; indeed, heterotropic regulation is not reported for D‐LDHs. Thus these authors completely dissociate the concept of allostery from the reaction mechanism. The structure of D‐LDHs is that of a homotetramer, and in the substrate‐bound form, intramolecular asymmetries are evident, consistent with the assigned reaction mechanism. While we applaud the attention given by Furukawa *et al*. to the reaction mechanism, we remark that the definition of allostery they adopt is peculiar: since D‐LDHs do not present (known) heterotropic regulation, they do not qualify for the definition given by Monod *et al*. [1]; and since they do not obey the MWC reaction mechanism, they do neither qualify for the definition given by Monod *et al*. [2]. D‐LDHs qualify for the sequential KNF reaction mechanism, but Koshland never used the term allostery to refer to his model [11]. The ‘sequential allosteric transitions’ referred to in the very title of the paper are events that neither belong to the MWC nor to the KNF model and testify that the term ‘allostery’ can be stretched in whatever direction the author feels appropriate.

## Allostery in monomeric proteins

Table 1 includes several monomeric proteins that present functional regulation and structure changes operated by heterotropic effectors, which have been described as instances of model‐independent allostery [94]. Monomeric proteins, however, are not described by Eqns (1), (2), (3), (4), (5), thus application of the concept of allostery to them requires further elaboration. Moreover, in the case of monomeric proteins the criteria we elaborated to assign the reaction mechanism are not applicable, as they rely mainly on the observation of homotropic cooperativity, and of intramolecular symmetry.

We begin our analysis with a word of caution: as Hans Frauenfelder showed many years ago [95], proteins are dynamic objects undergoing small scale structural fluctuations, which may be as subtle as movements of the side chains of single amino acids. This realizes a continuum of conformational isomers that is better described as a statistical distribution around a thermodynamic minimum, rather than as a tertiary structure change governed by the allosteric constant L. The vast majority of authors rightly excludes this phenomenon from the concept of allostery, and indeed this type of conformational fluctuations would occur in monomeric as well as in multimeric proteins, and in both the T and R states (if they exist in the protein under consideration) or in all the liganded and unliganded states.

Chi *et al*. [96] analyzed the reaction mechanism of a monomeric PDZ domain and considered the problem in some detail. They state: ‘Conformational selection and induced fit are two well‐known mechanisms of allosteric protein‐ligand interaction.’ Thus, as in the case of oligomeric proteins, the term allostery may cover at least two different reaction mechanisms, each with its own mathematical formulation. Unfortunately establishing the reaction mechanism of heterotropically regulated monomeric proteins is exceedingly difficult and has been possible only in a handful of cases. The criteria we underlined in Section ‘Assignment of the reaction mechanism to a list of allosteric proteins’ are not applicable to monomeric proteins, and one must rely on kinetic evidence; however kinetic evidence is conclusive only if the kinetic constants present some special relationships among each other [96, 97].

From the data available in the literature, we are not able to assign a reaction mechanism to the monomeric proteins listed in Table 1, thus in the present work we do not attempt their classification. Suffice it to say that if one wants to extend the concept of allostery to monomeric proteins, one finds there the same general problem we encountered in oligomeric proteins, i.e. allostery is an umbrella that covers more than a single reaction mechanism and cannot be defined by a single mathematical expression.

## Concluding remarks

Regulation of cell functions is key to physiology, and dysregulation is almost always a cause of disease. An important mechanism of regulation of protein (and, consequently, cell) function is allostery, which Monod called ‘the second secret of life’. The concept of allostery was initially formulated by J. Monod to describe heterotropic regulation of enzymes and other ligand binding proteins [1], and later in more stringent mechanistic terms [2]. Unfortunately, Monod's mechanism is not the only reaction mechanism capable of producing heterotropic regulation, and thus several different mathematical formulations are able to describe allostery; as a consequence many different types of allostery exist. This condition, which is *per se* undesirable, is further complicated by the fact that it is essentially unrecognized. Our analysis demonstrates that the concept of allostery has been extended to cover also cases unforeseen by classical thermodynamic models (e.g. monomeric proteins presenting Wyman's heterotropic linkage [87, 88], or proteins whose function is regulated by covalent chemical modification, which is thermodynamically irreversible, even though it can be undone enzymatically). We do not pretend to re‐define allostery; however, our work demonstrates that in its current usage this term includes many different reaction mechanisms, and does not have any precise and universally accepted meaning; at the very least, we should try to classify the many possible types of allostery.

It is unlikely that imprecise definitions are of help for science, e.g. if one tries to define structural parameters of ‘allosteric’ proteins, one ends up with very heterogeneous results; but we could obtain a reasonable description of some structural properties of allosteric proteins by grouping them according to their reaction mechanism [13]. The scope of this review is to warn researchers that the significance and usage of the term has become so broad that an effort is required to specify which type of allostery one is referring to. Some authors tried to address the point we analyze in this work, but unfortunately their conclusions disagree. Changeux and Edelstein [98] proposed that the vast majority of cases of allostery obey a population selection mechanism. Cui and Karplus [99] conducted a review analysis similar to the one presented here, but their examples are fewer and selected to illustrate some different possible cases, rather than to provide criteria for assigning the reaction mechanisms; their analysis includes proteins that obey a sequential reaction mechanism. Hilser *et al*. [100] proposed a comprehensive model capable of including conformation selection and sequential cooperativity, an approach opposite to the one we followed in this work: they try to unify, we try to separate and classify. Nussinov and co‐workers proposed the provocative, but imprecise idea that ‘allostery is an intrinsic property of all dynamic proteins’ [101], which is tantamount to say that allostery is the common property of ligand binding proteins that share none. Indeed, if all dynamic proteins were allosteric, we could dispose of the term allostery altogether. We suspect that this interpretation of allostery confuses two different concepts, whose boundary may indeed be blurry: the existence of two (or more) discrete thermodynamic states, as envisaged by the MWC model, and the existence of conformational ensembles first described by Frauenfelder and co‐workers. The latter phenomenon is indeed general, but is extended also to proteins whose function is not ligand binding, and to every state of truly allosteric proteins that may either obey the MWC or the KNF reaction mechanism. Clarity, once again, is provided by the mathematical definition, since the concept for Frauenfelder's conformational ensembles is described as the statistical distribution of minimally different conformers around an energy minimum, rather than via equations similar to Eqns (1), (2), (3), (4). A protein that obeys a two‐state reaction mechanism would have two widely separated minima (for the T and R state, respectively), with a distribution of minimally different conformers around each of them, but no intermediates in between.

An unexpected finding of the present analysis is that proteins that bind their ligands according to a sequential reaction mechanism and at the same time present heterotropic regulation are relatively uncommon, even though some cases listed as ‘undefined’ in Table 1 may qualify. We speculate that heterotropic regulation exerted at a tertiary structure level in an oligomeric cooperative enzyme is scarcely appealing from the viewpoint of physiology, because it is bound to produce incomplete inhibition or activation unless the concentration of the effector is high enough to saturate all the binding sites in the oligomer. Indeed, the cases of PGDH and the Arg repressor seem to confirm this speculation since in these cases effector binding is negatively cooperative but full inhibition does not require full saturation of the effector binding sites [13]; this however implies a reaction mechanism that involves a symmetric quaternary structural transition, albeit not requiring the free equilibrium between two different conformations. The KNF model does not forbid, but neither requires, quaternary structure changes, thus we consider this property atypical but not incompatible with respect to the premises of that model. The negative cooperativity of effector binding suggests that the quaternary structure change does not reflect an equilibrium between two conformations but is a case of ligand‐induced fit. We may summarize the preliminary results obtained from the comparative study of proteins that obey the KNF reaction mechanism and present heterotropic regulation as follows: (a) combination of the sequential reaction mechanism and heterotropic regulation is relatively uncommon; (b) a stoichiometry of one effector binding site per subunit is observed; (c) negative cooperativity in effector binding is frequent, as are subtle tertiary and quaternary structure changes; (d) often (but not necessarily always) the binding site of the effector lies in proximity of the intersubunit interfaces. Further work is required to validate these results.

## Conflict of interest

The authors declare no conflict of interest.

## Author contributions

AB designed the study and wrote the manuscript. VM and FA carried out the structural analyses.
